# Effects and feasibility of a multi-disciplinary orientation program for newly registered cancer patients: design of a randomised controlled trial

**DOI:** 10.1186/1472-6963-9-203

**Published:** 2009-11-11

**Authors:** Raymond Chan, Joan Webster, Linda Bennett

**Affiliations:** 1 Cancer Care Services, Royal Brisbane & Women's Hospital, Butterfield Street, Herston, QLD 4029, Queensland, Australia; 2 Centre for Clinical Nursing, Royal Brisbane & Women's Hospital, Butterfield Street, Herston, QLD 4029, Queensland, Australia; 3 School of Nursing and Midwifery, Queensland University of Technology, Kelvin Grove, QLD 4059, Australia

## Abstract

**Background:**

Diagnosis and treatment of cancer can contribute to psychological distress and anxiety amongst patients. Evidence indicates that information giving can be beneficial in reducing patient anxiety, so oncology specific information may have a major impact on this patient group. This study investigates the effects of an orientation program on levels of anxiety and self-efficacy amongst newly registered cancer patients who are about to undergo chemotherapy and/or radiation therapy in the cancer care centre of a large tertiary Australian hospital.

**Methods:**

The concept of interventions for orienting new cancer patients needs revisiting due to the dynamic health care system. Historically, most orientation programs at this cancer centre were conducted by one nurse. A randomised controlled trial has been designed to test the effectiveness of an orientation program with bundled interventions; a face-to-face program which includes introduction to the hospital facilities, introduction to the multi-disciplinary team and an overview of treatment side effects and self care strategies. The aim is to orientate patients to the cancer centre and to meet the health care team. We hypothesize that patients who receive this orientation will experience lower levels of anxiety and distress, and a higher level of self-efficacy.

**Discussion:**

An orientation program is a common health care service provided by cancer care centres for new cancer patients. Such programs aim to give information to patients at the beginning of their encounter at a cancer care centre. It is clear in the literature that interventions that aim to improve self-efficacy in patients may demonstrate potential improvement in health outcomes. Yet, evidence on the effects of orientation programs for cancer patients on self-efficacy remains scarce, particularly with respect to the use of multidisciplinary team members. This paper presents the design of a randomised controlled trial that will evaluate the effects and feasibility of a multidisciplinary orientation program for new cancer patients.

**Trial registration:**

Current Controlled Trials ACTRN12609000018213

## Background

There is a consensus that diagnosis and treatment of cancer contributes to psychological distress and anxiety among patients [1-3]. The literature has demonstrated that information can reduce distress by enhancing patients' sense of control. An enhanced sense of control, in turn, relieves anxiety and enhances management of illness [4]. Although the need for information exists across the continuum of cancer care [5,6] the anxiety can be particularly pronounced at the initial visit to a cancer centre [7,8]. Factors contributing to this anxiety and distress can be the lack of familiarity with the environment and with the care providers.

Researchers have investigated interventions with the aim of reducing the anxiety and distress levels among cancer patients [4,9]. One of these interventions includes orientation programs for patients who are newly registered to a cancer service. A study of 150 new cancer patients by McQuellon and research team reported that their orientation was effective in reducing anxiety, distress and depressive symptoms and enhanced knowledge and satisfaction of care [10]. In 2003, Gallant (2003) tested different methods of delivering an orientation program and found that it was more challenging to recruit patients for the face to face arms compared to the mail group [11]. The components of these orientation programs vary and generally include question and answer sessions, a clinic tour, description of procedures, provision of information. However, none of these studies have investigated the effects of orientation programs and were not conducted by a multidisciplinary team.

### Self-efficacy

In the population other than cancer patients, researchers have demonstrated that self-efficacy can be enhanced through orientation and educational programs, and that higher self-efficacy is related to improved health outcomes [12-15]. Self-efficacy is a construct of Bandura's social cognitive theory [16,17], defined as a person's belief that they can perform the specific behaviours necessary to achieve their goals. A person's sense of self-efficacy should lead to attempting and persisting when encountering difficulties; their confidence in or perception of their ability to carry out tasks directly relates to their likelihood of success [18]. A variety of research on cancer patients' self-efficacy has been conducted [19-23]. Self-efficacy is a potent factor which can affect the quality of life in cancer patients. Firstly, enhanced self-efficacy directly affects quality of life positively. Secondly, it reduces perceived stress and, in turn, increases quality of life [23]. That is, cancer patients with higher self-efficacy have lower level of anxiety and distress and higher quality of life [21,23,24]. Therefore, interventions with the aim of increasing ones' self-efficacy are recommended.

### Multidisciplinary team involvement

A systematic review reported that multidisciplinary care can result in positive patient outcomes [25], specifically in diagnosis and/or treatment planning [26-28], survival [29-31], patient satisfaction [26], communication and cooperation [32]. Although there is a vast body of literature supporting the positive outcomes as a result of multidisciplinary care, there is a paucity of evidence which demonstrates the importance of multidisciplinary involvement in conducting orientation programs for cancer patients. While evidence suggests that unfamiliarity with the care providers is one of the factors contributing to this anxiety and distress in this patient population [33], it is important to note that "care providers" should include every member that the patients will access care from during the continuum of cancer care. Orientation provided for cancer patients should no longer be limited to "meeting the oncologist". As the model of care has changed from a medical focus to a multidisciplinary model, health professionals conducting orientation programs should also respond to the change. However, the orientation programs documented in the literature are mainly conducted by one nurse coordinator, rather than a multidisciplinary team [10,33,34]. Therefore, research is needed to evaluate the effectiveness of an orientation program conducted by a multidisciplinary team.

### Objective of the study and the current paper

The main objectives of the study are

1. To develop an orientation program conducted by a multidisciplinary team for newly registered cancer patients in terms of its impacts on the patients' self-efficacy and anxiety.

2. To test the feasibility of a randomised controlled trial of an orientation program conducted by a multidisciplinary team for all newly registered cancer patients.

3. To evaluate the effectiveness of the program on patients' self-efficacy, anxiety, stress and informational awareness.

The current paper presents the design of the randomised controlled trial that will evaluate an orientation program conducted by a multidisciplinary team for newly registered cancer patients

## Methods

### Method

Randomised controlled trial will be used in this study.

### Ethics consideration

The study protocol has been reviewed and approved by the Royal Brisbane & Women's Hospital Health Service District Human Research Ethics Committee.

### Study population

All cancer patients newly registered at a tertiary cancer care centre in Brisbane, Australia, who meet inclusion criteria will be invited to participate in the trial. Patients who consent will be randomly allocated to either the control group, who will receive normal treatment education at the start of their treatment, or the intervention group, who will receive the normal treatment education and will also attend the Orientation Program education session conducted by the multi-disciplinary team, prior to starting their treatment.

#### Inclusion and exclusion criteria

Patients will be eligible if they consent and are undergoing a course of radiotherapy, chemotherapy or both; have a definitive diagnosis of cancer; are over 18 years of age; are able to read and write in English and are "well" enough to complete the questionnaire. Patients will be excluded if they have had previous cancer treatments.

### Randomisation and masking

Patients will be randomly allocated to either normal care (control group) with standard education provided by nurses at the first visit of treatment, or to the intervention group to participate in an extra orientation program.

#### Sequence generation

Blocked randomisation will be performed with a block size of four by a computer generated random number list prepared by an investigator who has no clinical involvement in the trial. Stratification by primary cancer diagnosis group will also be carried out.

#### Allocation concealment/implementation

After the research nurse has obtained the patient's consent, he/she will telephone a contact who is independent of the recruitment process for allocation concealment. Subsequently, baseline data will be collected. All questionnaires are self-administered by patients. Therefore, the research nurse will not have involvement in completing these questionnaires.

#### Blinding/masking

It is not possible to blind patients regarding the result of randomisation in this type of intervention. While it is not possible to blind the health professionals administering the orientation program, they will have no involvement in assessing and analysing the data.

### Intervention

The intervention is a Multidisciplinary Orientation Program. It will be run twice weekly in the oncology out-patients department, and comprises a 5 minute 'virtual hospital tour', a talk by a registered nurse giving an overview of potential side effects of radiation therapy and chemotherapy, and an opportunity to meet the health care team. The hospital tour highlights areas of the hospital that patients are likely to need during the course of their treatment, such as the pharmacies and the pathology department. It also will show practical areas such as the Food Court, Post Office and Automatic Teller Machines. Different parts of the Cancer Care Services building are shown - the check in desk, the different waiting areas, the day therapy unit and the radiation treatment floor. The side effects overview will include common side effects experienced by oncology patients and self care strategies. This talk was developed using the information currently given to patients in the respective treatment areas at the start of their treatment, but does not include details of specific treatment regimens.

The health care team consists of a nurse, a cancer care coordinator, a social worker, a occupational therapist, a physiotherapist, a speech pathologist and a discharge coordinator. The healthcare team each will have five minutes to give an overview of their role, how they may support the patients throughout their treatment and encounter at the cancer care centre and to give patients their contact details. The health care team each have their scripts and powerpoint slides prepared to ensure the consistency of each sessions. All patients attending the intervention will be given a 10-page booklet which contains the information given in the 'virtual tour' and expands on it, eg. prices for car parking, access to public transport, opening times for food outlets within the hospital, note space. It also has information about the role of each member of the health care team, telephone numbers for clinics, and tips for getting through treatment. At the end of the session there will be time for questions for both the nurse and the members of the healthcare team. Powerpoint presentation and a projector will be used to facilitate every section of the program. Scripts and notes have been designed and agreed by the health care team and will be used to ensure the consistency of each session.

### Measures

#### Anxiety

Anxiety will be measured using the trait anxiety version of the Spielberger's Trait Anxiety Inventory (STAI) and the Brief Profile of Mood States - Total Mood Disturbances Score (POMS-TMDS) [35-37].

The STAI was developed as a tool for investigating anxiety in normal adults, but has been used extensively for assessing anxiety in cancer patients [10,11,21,33]. The scale consists of 20 questions yielding a measure of state anxiety (S-Anxiety) which assesses how a patient feels generally. An extra 20 questions refer to trait anxiety (T-Anxiety) which refers to individual differences in "anxiety-proneness" [35]. The STAI demonstrated good psychometric properties [35]. Reliability has been substantiated by means of test-retest correlations (r = 0.65-0.86) and Cronbach's alpha (median coefficient of 0.90). Scores on the scales can range from 20-80, with higher scores indicating more symptoms of anxiety [21].

The POMS-TMDS is a shorter version of the 65 item Profile of Mood States (POMS) [37]. It consists of 11 items that yield a total mood disturbance score that correlates highly (r = 0.93) with the longer POMS which has been utilised extensively for the cancer population [10,11,33,38]. Reliability and validity testing has been carried out successfully in patient with cancer [39]. Permission for use has been granted by the author.

#### Self -efficacy

The communication and attitudinal self-efficacy scale for cancer (CASE-cancer) has been selected to measure self-efficacy in the patients [19]. The CASE-cancer is a psychometrically sound tool with high internal consistency and construct validity. The scale items are performed similarly across literacy levels. It is also recommended for testing the effect of interventions to improve communication, education and involvement in cancer [19]. Permission for use has been granted by the author.

#### Evaluation of Oncology Clinic Questionnaire (EOCQ)

The research team has designed a tool which will specifically investigate whether patients have received particular information prior to their first treatment day (Yes/No). It has included a list of information that the research team believes to be important for patients before they commence their cancer treatment. This tool will be administered at Time 2 and Time 3.

### Data collection

Participants will be asked to complete a series of questionnaires at the time of recruitment (T1), on the day of first treatment (T2), and on the seventh day of treatment (T3) which will measure patients' self-efficacy (CASE) and anxiety (STAI and POMS) at the three time points. Additionally, demographic information will be collected at T1 and the EOCQ will be collected at T2 and T3.

### Sample size and power

A sample size of at least 375 in each arm of the study would be sufficient to achieve a power of 90% using 95% of confidence interval to detect a difference in the anxiety and self-efficacy scores between control group and intervention group. Assuming that approximately 25% fail to consent and a further 10% will be lost to follow-up; an additional 132 in each group will be required and the final sample will require 1,014 patients. In order to evaluate the research design and process, the current proposal is for a pilot study with 10% of the final sample (ie 102 patients, 51 in each arm). According to the statistics of RBWH Cancer Care Services, 274 new cancer outpatients receiving treatment each month. Thus, the sample size proposed is achievable over a period of 12 weeks based on our conservative estimation.

### Analysis

All data will be analysed according to intention-to-treat principle. Means, standard deviations will be used to summarise overall and within subgroups defined by the range of medical, sociological, and other risk factors collected at baseline. Mean scores of POMS, STAI and CASE-cancer will be compared initially across intervention and control groups using ANOVA (repeated measures) to analyse the effect between groups over time (or Friedman test if normality is not a valid assumption). Success of randomisation will then be considered by cross-tabulating trial group with the range of factors. Any found to be in substantial imbalance will be included in multivariable linear regression models, via forced entry, to adjust the crude means and these will be reported with 95% confidence intervals. Analyses will use the SPSS (Version 16) package and a generalised linear modelling framework.

## Discussion

An orientation program is a common health care service provided by cancer care centre for new cancer patients. Such programs aim to give information to patients at the beginning of their encounter at a cancer care centre. It is clear in the literature that interventions that aim to improve self-efficacy in patients may demonstrate potential health outcomes. Yet, evidence on the effects of orientation programs for cancer patients on self-efficacy remains scarce, particularly with respect to the use of multidisciplinary team members. This paper presents the design of a randomised controlled trial that will evaluate the effects and feasibility of a multidisciplinary orientation program for new cancer patients.

## Competing interests

The authors declare that they have no competing interests.

## Authors' contributions

RC, JW contributed to the conception and design of the study. All authors were involved in the preparation of the manuscripts. All authors read and approved the final manuscript.

## Pre-publication history

The pre-publication history for this paper can be accessed here:

http://www.biomedcentral.com/1472-6963/9/203/prepub

## References

[B1] Hefez A Bager L Arison Z Anxiety in cancer patients: A pilot study Israel Journal of Psychiatry and Related Science 1982 19 303 313 7184900

[B2] Holland J Psycho-oncology: overview, obstacles, and opportunities Psycho-oncology 1992 1 1 13 10.1002/pon.2960010103 29749682

[B3] Sussman N Reactions of patients to the diagnosis and treatment of cancer Anti-Cancer Drugs 1995 1 4 8 10.1097/00001813-199502001-00002 7749169

[B4] Chelf J Agre P Axelrod A Cheney L Cole D Conrad K Cancer related patient education: An overview of the last decade of evaluation and research Oncology Nursing Forum 2001 28 1139 1147 11517847

[B5] Luker K Beaver K Leinster S Owens R Information needs and sources of information for women with breast cancer: A follow-up study Journal of Advanced Nursing 1996 23 487 495 10.1111/j.1365-2648.1996.tb00010.x 8655823

[B6] White N Given B Devoss D The advanced practice nurse: Meeting the information need of the rural cancer patients Journal of cancer education 1996 11 203 209 8989633 10.1080/08858199609528429

[B7] Mohide E Whelan T Rath D Gafni A Willan A Czukar D A randomised trial of two information packages distributed to new cancer patients before their initial appointment at a regional cancer centre British Journal of cancer 1996 73 1588 1593 8664135 10.1038/bjc.1996.299 PMC2074537

[B8] Farber J Weinerman B Kuypers J Psychosocial distress in oncology outpatients Journal of Psychosocial Oncology 1992 2 109 118 10.1300/J077v02n03_09

[B9] Devine E Westlake S The effects of psychoeducational care provided to adults with cancer: Meta-analysis of 116 studies Oncology Nursing Forum 1995 22 9 1369 1381 8539178

[B10] McQuellon R Wells M Hoffman S Craven B Russell G Cruz J Hurt G DeChatelet P Andrykowski M Savage P Reducing distress in cancer patients with an orientation program Psycho-oncology 1998 7 207 217 10.1002/(SICI)1099-1611(199805/06)7:3<207::AID-PON304>3.0.CO;2-T 9638782

[B11] Deshler A Fee-Schroeder K Dowdy J Mettler T Novotny P Zhao X Frost M A patient orientation program at a comprehensive cancer centre Oncology Nursing Forum 2006 33 3 569 578 10.1188/06.ONF.569-578 16676013

[B12] Lorig K Robert L Chastian M Ung E Shoor S Holman H Development and evaluation of a scale to measure perceived self-efficacy in people with arthritis Arthritis and Rheumatism 1989 32 1 37 44 10.1002/anr.1780320107 2912463

[B13] Pellino T Tluczek A Collins M Trimborn S Norwick H Engelke Z Broad J Increasing self-efficacy through empowerment: preoperative education for orthopaedic patients Orthopaedic Nursing 1998 17 4 48 59 10.1097/00006416-199807000-00009 9814337

[B14] Clark N Janz N Dodge J Sharpe P Self-regulation of health behavior: the "take PRIDE" program Health Education Quarterly 1992 19 3 341 354 1517097 10.1177/109019819201900306

[B15] Boehm S Coleman-Burns P Schlenk E Funnell M Parzuchowski J Powell I Prostate cancer in African American Men: Increasing knowledge and self-efficacy Journal of community health nursing 1995 12 3 161 169 10.1207/s15327655jchn1203_4 7561994

[B16] Bandura A Self-efficacy: Toward a unifying theory of behaviour change Psychological Review 1977 84 191 215 10.1037/0033-295X.84.2.191 847061

[B17] Bandura A Social Foundations of Thought and Action 1986 Englewood Cliffs: NG: Prentice-Hall

[B18] Shortridge-Baggett L Lenz E Self-efficacy in Nursing: Research and Measurement Perspectives 2002 New York: Springer Publishing Company

[B19] Wolf M Chang C Davis R Makoul G Development and validation of the Communication and Attitudinal Self-efficacy scale for cancer (CASE-cancer) Patient Education and Counselling 2005 57 333 341 10.1016/j.pec.2004.09.005 15893217

[B20] Han W Collie K Koopman C Breast cancer and problems with medical interactions: relationships with traumatic stress, emotional self-efficacy, and social support Psycho-oncology 2005 14 4 318 330 10.1002/pon.852 15386762

[B21] Porter L Keefe F Garst J McBride C Baucom D Self-efficacy for managing pain, symptoms, and function in patients with lung cancer and their informal caregivers: Associations with symptoms and distress Pain 2008 137 306 315 17942229 10.1016/j.pain.2007.09.010 PMC2522367

[B22] Beckham J Burker E Self-efficacy and adjustment in cancer patients: A preliminary report Behavioural Medicine 1997 23 3 138 144 10.1080/08964289709596370 9397286

[B23] Kreitler S Peleg D Ehrenfeld M Stress, self-efficacy and quality of life in cancer patients Psycho-oncology 2007 16 329 341 10.1002/pon.1063 16888704

[B24] Cunningham A Lockwood G Cunningham J A relationship between perceived self-efficacy and quality of life in cancer patients Patient Education and Counseling 1991 17 71 78 10.1016/0738-3991(91)90052-7 1997998

[B25] Wright F Devito C Hunter A Multidisciplinary cancer conference: A systematic review and development of practice standards European Journal of Cancer 2007 43 1002 1010 10.1016/j.ejca.2007.01.025 17329094

[B26] Gabel M Hilton N Nathanson S Multidisciplinary breast cancer clinics. Do they work? Cancer 1997 79 12 2380 2384 10.1002/(SICI)1097-0142(19970615)79:12<2380::AID-CNCR12>3.0.CO;2-N 9191526

[B27] Chang J Vines E Bertsh H Fraker D Czerniecki B Rosato E The impact of a multidisciplinary breast cancer centre on recommendations for patient management: The University of Pennsylvania experience Cancer 2001 91 7 1231 1237 10.1002/1097-0142(20010401)91:7<1231::AID-CNCR1123>3.0.CO;2-K 11283921

[B28] Santosa J Schwertner B Coleman R Hannigan E Tumor board in gynecologic oncology International Journal of Gynecologic Cancer 2004 14 2 206 209 10.1111/j.1048-891X.2004.014200.x 15086716

[B29] Junor E Hole D Gillis C Management of ovarian cancer: referral to a multidisciplinary team matters British Journal of cancer 1994 70 2 363 370 8054286 10.1038/bjc.1994.307 PMC2033481

[B30] Sainsbury R Haward B Rider L Johnston C Round C Influence of clinician workload and patterns of treatment on survival from breast cancer Lancet 1995 345 8960 1265 1270 10.1016/S0140-6736(95)90924-9 7746056

[B31] Birchall M Bailey D King P Effect of process standards on survival of patients with head and neck cancer in the south and west England British Journal of cancer 2004 91 8 1477 1481 15467772 10.1038/sj.bjc.6602118 PMC2409928

[B32] Smith D Davis S Polissar L The hospital cancer program: its impact on care of the rural cancer patients The American Surgeon 1979 45 11 730 737 517872

[B33] Wells M McQuellon R Hinkle J Cruz J Reducing anxiety in newly diagnosed cancer patients- a pilot program Cancer practice 1995 3 2 100 104 7704066

[B34] Gallant M Coutts L Evaluation of an oncology outpatient orientation program: Patient satisfaction and outcomes Supportive care in Cancer 2003 11 800 805 10.1007/s00520-003-0543-8 14564644

[B35] Speilberger C Manual for the State-Trait Anxiety Inventory (Form Y) 1983 Palo Alto, CA: Consulting Psychologists

[B36] McNair D Lorr M Droppleman L Profile Mood States manual 1971 Sandiego, CA: Educational and Testing Service

[B37] Cella D Jacobsen P Orav E Holland J Siberfarb P Rafla S A brief POMS measure of distress for cancer patients Journal of Chronic Diseases 1987 40 939 942 10.1016/0021-9681(87)90143-3 3611291

[B38] Siston A List M Daugherty C Banik D Menke C Cornetta K Larson R Psychosocial adjustment of patients and caregivers prior to allogenic bone marrow transplantation Bone Marrow Transplantation 2001 27 1181 1188 10.1038/sj.bmt.1703059 11551029

[B39] Curran S Andrykowski M Studts J Short form of the Profile of mood states (POMS-SF): Psychometric information Psychological Assessment 1995 7 1 80 83 10.1037/1040-3590.7.1.80

